# Socioeconomic and geographic inequalities in adolescent smoking: A multilevel cross-sectional study of 15 year olds in Scotland

**DOI:** 10.1016/j.socscimed.2014.02.016

**Published:** 2014-04

**Authors:** K.A. Levin, R. Dundas, M. Miller, G. McCartney

**Affiliations:** a CAHRU, School of Medicine, University of St Andrews, Medical and Biological Sciences Building, North Haugh, St Andrews KY16 9TF, UK; b University of Glasgow, UK; c University of St Andrews, UK; d NHS Health Scotland, UK; e Ludwig Boltzmann Institute for Health Promotion Research, Vienna, Austria

**Keywords:** Scotland, Urban–rural, Smoking, Adolescent, Multilevel modelling, Socioeconomic inequalities

## Abstract

The objective of the study was to present socioeconomic and geographic inequalities in adolescent smoking in Scotland. The international literature suggests there is no obvious pattern in the geography of adolescent smoking, with rural areas having a higher prevalence than urban areas in some countries, and a lower prevalence in others. These differences are most likely due to substantive differences in rurality between countries in terms of their social, built and cultural geography. Previous studies in the UK have shown an association between lower socioeconomic status and smoking. The Scottish Health Behaviour in School-aged Children study surveyed 15 year olds in schools across Scotland between March and June of 2010. We ran multilevel logistic regressions using Markov chain Monte Carlo method and adjusting for age, school type, family affluence, area level deprivation and rurality. We imputed missing rurality and deprivation data using multivariate imputation by chained equations, and re-analysed the data (*N* = 3577), comparing findings. Among boys, smoking was associated only with area-level deprivation. This relationship appeared to have a quadratic S-shape, with those living in the second most deprived quintile having highest odds of smoking. Among girls, however, odds of smoking increased with deprivation at individual and area-level, with an approximate dose–response relationship for both. Odds of smoking were higher for girls living in remote and rural parts of Scotland than for those living in urban areas. Schools in rural areas were no more or less homogenous than schools in urban areas in terms of smoking prevalence. We discuss possible social and cultural explanations for the high prevalence of boys' and girls' smoking in low SES neighbourhoods and of girls' smoking in rural areas. We consider possible differences in the impact of recent tobacco policy changes, primary socialization, access and availability, retail outlet density and the home environment.

## Highlights

• We describe adolescent smoking, adjusting for individual and area-level deprivation and rurality.
• Boys living in the second most deprived quintile have highest odds of smoking.
• Among girls, odds of smoking increases with deprivation at individual and area-level.
• Girls' smoking prevalence is higher in remote rural than in urban Scotland.
• Multiple imputation supports the findings of complete-case analysis.

## 1 Introduction

Smoking is a major risk factor for lung cancer, high blood pressure, ischaemic heart disease, stroke, emphysema, and asthma. Smoking during adolescence is of particular interest because it is associated with other health damaging behaviours such as alcohol and cannabis use, fighting and unprotected sex (CDC, 1994). This is a life stage where many health behaviours are initiated, often tracking into adulthood (Jarvis, 2004). In the US, for example, 80% of adult smokers begin smoking before the age of 18 (Campaign for Tobacco-Free Kids, 2013). Furthermore, substance use during adolescence has a greater negative impact on the brain than in adulthood, increasing the risk of addiction, and negatively affecting memory, concentration and judgement (Chambers et al., 2003; Crews et al., 2007). The negative effects of smoking to the individual smoker are further compounded by the fact that young people who smoke are more likely to be exposed to secondhand smoke through their peers and parents, as smokers are more likely to have friends who smoke (West and Michell, 1999) and are more likely to have one or more parents who smoke (Gilman et al., 2009). Moreover, exposure to secondhand smoke, after controlling for adolescents' own smoking, is linked to asthma, respiratory problems and arterial thickening (Kallio et al., 2010; Vork et al., 2007). Reducing smoking in adolescence is therefore not only beneficial for individuals but also for the overall population.

Although smoking rates in Scotland have reduced, socio-economic inequalities in smoking prevalence rates must be tackled if Scotland is to achieve its ambition of becoming smoke-free by 2036 (Scottish Government, 2013). The 2011 Scottish Household Survey illustrates that while smoking prevalence among Scottish adults has fallen from 31% in 1999 to 23.3% in 2011, rates remain disproportionately high among those living in areas of high deprivation (i.e. 40% in the most deprived compared to 11% in the least deprived communities) (Scottish Government, 2012a). Among 15 year olds, inequalities in smoking are dependent on the measure used; while 23% of non-smokers live in the least deprived quintile compared with 14% in most deprived quintile, 22% of occasional smokers live in the least deprived quintile compared with 10% in the most deprived, and 17% of regular smokers live in the least deprived and 17% in the most deprived quintiles (Black et al., 2011).

Studies of socioeconomic and geographic inequalities are important, firstly, because as health improves, as it has done over recent decades in Scotland, greater improvements are often observed among some members of the population than others (Wagstaff, 1991). This was also seen in an evaluation of health publicity, which showed no decrease in smoking among British adults of lowest social class (Townsend et al., 1994). Ignoring inequalities may lead us to the incorrect conclusion that the population as a whole is improving. Secondly, by identifying subgroups within the population whose health is particularly poor or particularly good, we may progress to identify associated modifiable risk factors, a first step in putting interventions in place for those at the greatest risk.

Internationally, a larger number of country-specific studies have considered urban–rural differences in adolescent smoking. However, the findings are at odds. A review of psychosocial correlates with adolescent smoking concluded that the relationship with rural residence was ‘undecided’, with a higher prevalence found in rural tobacco-producing areas of the US and in urban Sri Lanka and Finland. A further two studies, in Iceland and New Zealand, which were included in the review, showed no relationship at all (Tyas and Pederson, 1998). Subsequent studies in China, Slovakia, Germany, Greece, Peru and the Sudan have shown a higher prevalence of smoking among urban adolescents (Ho et al., 2010; Hujova and Lesniakova, 2011; Idris et al., 1998; Robinson et al., 2011; Spyratos et al., 2012; Volzke et al., 2006), although in Argentina, Taiwan, and Korea smoking was more prevalent among rural adolescents (Mulassi et al., 2010; Chang et al., 2011; Park, 2010). Furthermore, a study in Lithuania found the relationship to be dependent on gender; while boys living in rural areas smoked more frequently, girls living in urban areas did so (Zaborskis et al., 2009). Studies carried out in the US appear to contradict one another (Evers et al., 2001; Lutfiyya et al., 2008; Mistry et al., 2011).

Differences in association by country may be due to differences in comparability of studies caused by the indicators of rurality used (Brady and Weitzman, 2007), or indeed smoking eg occasional versus regular (Black et al., 2011), or may be due to substantive differences in rurality between countries; rural lifestyles in a highly urbanised country such as Taiwan (population density of 645 per Km^2)^) is likely differ from that of a country such as Peru (population density of 23 per Km^2^). In particular there are cultural and socioeconomic differences in rural areas of low, middle and high income countries and those which have experienced a recent transition from one classification to another. Country-specific results are therefore primarily relevant to those countries with a similar social, built and cultural geography.

Scotland has a population of approximately 5,250,000 with a landmass of 78,772 km^2^. However, most of the population of Scotland resides in the central belt which includes the two largest cities, Glasgow and Edinburgh, and several other large towns. The Highlands and Islands, home to 7% of the Scottish population, makes up over 60% of Scottish landmass, with a resulting sparse population density of 8 people per square kilometre. These large differences in geography make the study of urban–rural differences in Scotland particularly interesting. Previous research of adult health has shown less favourable outcomes in remote rural Scotland; higher rates of suicide (Levin and Leyland, 2005) and ischaemic heart disease following discharge from hospital (Levin and Leyland, 2006a), more severe injuries due to road traffic accidents (Weiss et al., 2001) and more advanced stages of cancer at diagnosis (Campbell et al., 2001), after adjustment for socioeconomic status.

Adjustment for SES is particularly important in the study of urban-rural health inequalities because of sociodemographic differences in Scotland's geographies (Bishop et al., 2004; Levin and Leyland, 2006b). Rural areas, and particularly rural areas located within a 30 min drivetime from urban centres, also known as ‘accessible rural’ areas, have lower rates of deprivation. Unadjusted geographic analyses may therefore be confounded by deprivation. Conversely, adjustment for rurality is therefore relevant in the study of socioeconomic inequalities. Although various socioeconomic and geographic measures and proxy measures have been analysed at individual and higher levels in association with adolescent smoking in Scotland (Black et al., 2011; Corbett et al., 2005), no previous study has analysed these simultaneously.

The aim of the current study is to describe adolescent smoking behaviour across the urban–rural spectrum, and by socioeconomic status. The objectives are to 1. examine urban–rural differences in adolescent smoking for a number of different smoking measures, 2. quantify socioeconomic inequalities, by measuring the independent effect of individuals' affluence, school type and area-level deprivation, 3. investigate whether socioeconomic inequalities differ by rurality.

## 2 Methods

### 2.1 Study design

This paper examines Scottish data from the 2010 Health Behaviour in School-aged Children (HBSC) survey, a WHO collaborative cross-national study conducted in 43 countries in Europe and North America (Currie et al., 2009). The population was stratified by education authority and school type, defined as state-funded or independent, and a nationally representative sample was selected using systematic random sampling. Using passive parental consent (i.e. if parents did not state otherwise their children took part in the survey), pupils in Secondary 4 (S4), aged approximately 15.5 years, received questionnaires in school between January and March. The paper-based questionnaire was completed anonymously in class under teacher supervision. The research protocol was approved by the University of Edinburgh's School of Education Ethics Committee.

The 2010 HBSC Scotland survey sample of S4 pupils was boosted in order to be representative of urban and rural Scotland. Table 1 provides the classification system used to define the different geographies of Scotland. Urban Scotland makes up the greatest proportion of the population and is therefore generally well represented within the HBSC Scotland survey. The boosted sample of classes was selected randomly within each sampling frame, defined by rurality classification, assigned by school postcode. The samples were boosted with the aim of achieving a minimum of 300 children within each rurality classification to give 95% confidence intervals of ±6% around a proportion of 65% (for 15 year olds, the majority of variables saw proportions greater than 65% or smaller than 35% in the 2006 HBSC survey (Currie et al., 2008a)) and a design factor of 1.2, an adjustment made to account for fact that the data are clustered by school.

### 2.2 Outcome variables

Smoking nicotine was examined in the study, using 4 outcomes: Tried smoking, Current smoking, Weekly smoking and Daily smoking. Tried smoking was measured using the following item: Have you ever smoked tobacco? (At least one cigarette, cigar or pipe) with response options ‘Yes’/‘No’. The item: How often do you smoke tobacco at present? with response options: ‘Every day’/‘At least once a week, but not every day’/‘Less than once a week’/‘I do not smoke’ was used to create binary measures: Current Smoking (‘I do not smoke’ versus all other responses), Weekly Smoking (‘Every day’/‘At least once a week’ versus ‘Less than once a week’/‘I do not smoke’) and Daily smoking (‘Every day’ versus all other responses).

### 2.3 Explanatory variables

Young people's age was included in analysis. School type (state or independent) was also included. The Family Affluence Scale (FAS) (Currie et al., 2008b) was calculated using responses to the following questions: Does your family have a car or van? (no/yes, one/yes, two or more); Do you have your own bedroom to yourself? (no/yes). During the past 12 months, how many times did you travel away on holiday with your family? (not at all/once/twice or more). How many computers (PCs, Macs or laptops) does your family own? (none/one/two/more than two). The items were combined using categorical principal components analysis to produce tertiles of low, medium and high family affluence.

Deprivation at the area level was assigned to individual child's home postcode, requested by the survey, using the 2012 Scottish Index of Multiple Deprivation (SIMD), a standardised measure of deprivation at the ‘data zone’ small-area level (Scottish Government, 2012b). The results presented were for relative deprivation using quintiles of the SIMD's income deprivation domain, as the overall SIMD included within it domains related to health, as well as access to services, a possible proxy for rurality. Analyses were also carried out using the overall SIMD quintiles with no difference in the overall conclusions. Rurality was included as a categorical variable as defined by the 2008 Scottish Government urban–rural classification (Scottish Government, 2008), an earlier version of which has been used previously in similar analyses (Levin and Leyland, 2005, 2006a; Levin et al., 2010), again assigned to the child's postcode.

### 2.4 Missing data

Of the original 3577 young people surveyed, 885 (25%) were excluded due to missing postcode information (which meant both rurality and SIMD were unable to be assigned), 54% boys and 46% girls. The final dataset had 2692. Among those excluded, smoking was more prevalent; while, in the study group, 39% had tried smoking, 48% of those who were not included had. Similarly, 16% were currently smoking in the study group, compared with 27% of those excluded, while 12% were weekly smokers compared with 21%, and 9% smoked daily compared with 15% of those excluded. However, there appeared to be little response bias by affluence with 34% of those excluded having low FAS, 34% middle FAS and 32% high FAS. A further 8 (0.3%), had missing responses to ever tried smoking, while 9 (0.3%) had missing responses to current, weekly and daily smoking outcomes.

To avoid exclusion and maximise the power of the study, multiple imputation by chained equation (MICE) was carried out in STATA version 11, to impute missing information for indicators of area level deprivation and rurality (Azur et al., 2011). In addition to predictor and outcome variables, we included measures associated with area level deprivation and rurality in the imputation model, in order to minimise bias, as recommended: perceived safety of local area, good places to go locally, able to trust people locally, litter in local neighbourhood, time taken to get to school, method of travel to school, reported physical activity and education authority. We generated twenty imputated datasets to obtain information about the rurality and SIMD variables.

### 2.5 Statistical analysis

Preliminary analyses described the data, presenting prevalence of smoking behaviour by rurality. These were compared using Chi-square tests as a preliminary analysis. The dataset was stratified by gender as there are known gender differences in adolescent smoking and associated factors (Bauer et al., 2007). Logistic multilevel regression models were then fitted for each of the smoking outcome variables, using Markov chain Monte Carlo methods in MLwiN (Rasbash et al., 2009) and fixed and random parameter estimates were tabulated. Wald tests were carried out to identify significance of parameter estimates. Estimates reported in the results are based on a chain of length of 50,000 following a burn-in of 15,000. The models had three levels: education authority (*n* = 32), school (*n* = 168), and individual child (*n* = 2692 in complete-case dataset, *n* = 3577 in imputed dataset). The models for all boys' outcomes and for all girls' outcomes with the exception of ‘Daily smoker’ were fitted, adjusting for age, sex, school type (state or independent), FAS, SIMD quintiles and rurality, to describe differences by geography. The model for ‘Daily smoker’ girls was fitted adjusting for age, FAS, SIMD quintiles and rurality only. A second set of models also adjusted for an interaction between rurality and SES, i.e. individual (FAS) and area deprivation (SIMD quintiles). When the boys' and girls' datasets were merged and modelled with gender interactions for each of the variables, the findings supported the gender differences described below. These models estimates are not presented in this paper but are available from the corresponding author on request. The models were then re-analysed using the imputed datasets and combined using Rubin's Rule (Rubin, 1997). Results for complete-case and imputed datasets were compared and discussed.

## 3 Results

Table 2 describes smoking prevalence by a number of variables. Girls were more likely to have tried smoking, be current smokers and were more likely to smoke weekly, and daily, than boys (*p* < 0.05). Although “ever tried smoking” did not differ by family affluence, young people from low affluent backgrounds were more likely to be current smokers, and to smoke weekly and daily. Young people attending independent schools were also less likely to be daily smokers (*p* < 0.001). Compared with those from the 4 Cities, young people from remote rural areas were more likely to have tried smoking (*p* = 0.002), and be current smokers (*p* = 0.04). Prevalence of smoking, however was not highest among those living in the most deprived quintile but in the second most deprived. SIMD 5 (least deprived) had significantly lower proportions of those who had tried smoking and of current smokers than SIMD 2, and significantly lower proportions of daily smokers and of weekly smokers than SIMD 1 and SIMD 2.

When the data were modelled and all factors adjusted for simultaneously, boys' smoking was not associated with individual's family affluence, school type attended or rurality (Table 3). Area-level deprivation, however, was significant in the model for all outcomes apart from daily smoking, under the joint chi-squared test. The relationship between smoking and deprivation was a quadratic one, with the highest odds of smoking for SIMD 2 or SIMD 3 relative to SIMD 5 (least deprived), for all outcomes. Ever tried smoking in particular appeared to have an S-shaped relationship with deprivation. School level variance was significant for current smoking only, under a one-sided test.

When girls' data were modelled, family affluence was associated with current smoking only; odds of smoking for those with low family affluence were 1.70 (1.15, 2.52) those with high FAS (Table 4). Area-level deprivation also had an independent significant effect with increased odds of smoking with increasing deprivation. Rurality was significantly associated with girls' smoking with higher odds of smoking among those from remote rural areas relative to urban adolescents for all outcomes. Girls living in accessible rural areas also had increased odds of daily smoking (OR = 2.58 (1.24, 5.36)) while those who lived in remote towns were most likely to have ever tried smoking (OR = 2.48 (1.40, 4.39)). Unexplained variance at the school level remained significant for outcomes “ever tried smoking” and “current smoking”, even after adjustment for all variables. Interactions between rurality and SIMD or FAS were not significant for any outcome for boys and girls. A random slope for rurality or deprivation at the school level was also not significant for boys' “current smoking” or girls' “ever tried smoking”.

Imputation of missing data increased smoking frequencies for boys and girls. The analyses conducted using the imputed datasets corroborated the findings of the analyses using the complete-case datasets, with a few minor exceptions. Table 5 presents a summary of the significant fixed and random effects, following analyses of the imputed datasets. In particular, effect sizes of living in remote towns and rural areas were smaller for the imputed dataset, but remained significant nonetheless suggesting higher odds of smoking in rural areas and remote towns. FAS was significant for all but tried smoking for girls after imputation. Random effects showed greater variation at the school level for the imputed datasets, particularly for girls. Associations between smoking outcomes and SIMD remained approximately the same for both boys and girls following imputation of the missing data.

## 4 Discussion

### 4.1 Geographic and socioeconomic differences in smoking

The study found a relationship between smoking and rurality and smoking and area-level deprivation, after adjustment for individual SES. However, analyses showed that sociodemographic correlates of adolescent smoking differ by gender, in line with previous studies of adult smoking behaviour (Bauer et al., 2007). These differences were observed before and after imputation of missing data. Among boys, smoking was associated only with area-level deprivation. This relationship appeared to have a curvilinear shape, with those living in the second most deprived quintile having highest odds of smoking for all smoking outcomes. Among girls, however, odds of smoking increased with deprivation at individual and area-level, with an approximate dose–response relationship for both, and these effects were independent of one another.

These findings are in accordance with some results reported previously by SALSUS, though not all (Black et al., 2011; Corbett et al., 2005). SALSUS also found that regular smokers were more likely to live in deprived areas, where also a greater number of cigarettes were smoked (Black et al., 2011), and described a stronger relationship between SIMD and regular smoking for girls than boys. However, for occasional smoking no discernible pattern was seen for either. The current study adjusted for FAS and SIMD simultaneously, showing that area-level deprivation was more relevant than affluence at the individual level.

The current study also shows that odds of smoking did not vary by rurality for boys for all four smoking outcomes, while odds of smoking were higher for girls living in remote rural Scotland than for those living in the 4 Cities, after adjustment for deprivation. Odds of daily smoking were also higher in accessible rural areas, while odds of ever having tried smoking were higher in remote towns. The only previous study of urban-rural differences in adolescent smoking in Scotland, carried out by SALSUS in 2004, did not adjust for SES and classified rurality by school (Corbett et al., 2005). As many rural children commute to school, particularly secondary school, many may have been misclassified using this method.

### 4.2 Tobacco policies in Scotland

Recent policy changes related to smoking include the introduction of larger hard-hitting health warnings on cigarette packets in 2003, followed by an amendment to the Tobacco Products Regulations 2007 legislation requiring picture warnings and a smoking ban in public places instated in 2006 (Smoking, Health and Social Care (Scotland) Act, 2005). Evaluation of the smoking ban in Scotland, found a significant increase in smoking cessation among adults (Fowkes et al., 2008), while it is believed that health warnings and pictorial imagery have contributed to the de-normalisation of tobacco smoking (Wardel et al., 2010).

However, the impact of smoking cessation policies, health publicity and tobacco retailing and advertising legislation is dependent on individual variables, such as SES (Townsend et al., 1994; Wardel et al., 2010), and higher-level factors such as social networks and characteristics of the neighbourhood context (Pearce et al., 2012), resulting in increased clustering of smoking in disadvantaged groups and places. Evaluation of the smoking ban in Scotland, immediately following implementation, found a persisting socioeconomic pattern, with children from lower SES households continuing to have higher exposure to secondhand smoke (Akhtar et al., 2010), and recommended that inequalities in future smoking behaviour be monitored, allowing time for the ban to have a longer term impact.

Although the Scottish Tobacco Act, 2010 introduced an increase in the legal age for purchasing tobacco in Scotland (from 16 to 18 years), younger adolescents find ways around this law (Robinson and Amos, 2010). Smoking policies aimed at adults are therefore also likely to impact on adolescent consumption, not only directly, through product regulation and purchasing legislation, but also indirectly, through changes in societal norms and social modelling of older peers and parents (Gilman et al., 2009; Mercken et al., 2012). The findings of the current study suggest that SES patterns observed among adults immediately following the ban are also seen in the adolescent population four years later. Geographic patterns of adolescent smoking among girls however are in contrast to adult findings, which suggest lower prevalence of smoking in rural areas, albeit at a higher aggregate level (Whyte et al., 2007).

### 4.3 Socialization and contextual differences in adolescent smoking

The three primary sources of socialization, peers, school and the family, are the most influential factors of an individual's social behaviour, including risk behaviours such as smoking. Higher prevalence of smoking among rural girls and young people living in deprived areas could therefore be due to cultural differences in societal norms within one or more of these social contexts. For example, it may be that prevalence of smoking is higher among rural mothers, or that female friendship groups have a stronger influence in smaller communities. Oetting et al. (1998) discuss ways in which neighbourhoods and their community characteristics influence sources of primary socialization through the strengthening and weakening of bonds.

The school level was significant, particularly for girls in this study even after adjustment for all factors. School clustering of smokers has been shown previously. This finding suggests significant differences between schools in the prevalence of smokers, and indicates that there is a clear role for schools to play in preventing the uptake of and reduction of smoking among young people but especially girls. However, this was true for both rural and urban areas, as the school effect did not differ by rurality; schools in rural areas were no more or less homogenous than schools in urban areas in terms of smoking prevalence.

### 4.4 Environmental factors and contextual differences in adolescent smoking

Aside from primary socialization agents, there may be a separate influence of the environment through community and locality on health behaviours. Environmental factors include access and availability, and social norms shaped by the rural environment (Veitch, 2009). There should, however, be less access to/availability of cigarettes in rural areas, as distance to nearest shop and lack of anonymity of under-age customers should present greater barriers (Kloep et al., 2001). Moreover, locational access to tobacco retail outlets has not been associated with smoking behaviour among adults (Pearce et al., 2009), while among adolescents, likelihood of smoking initiation is increased by a higher density of retail outlets surrounding schools (Henriksen, 2012). As density of retail outlets in rural areas is likely to be lower, this does not explain the finding that girls in rural areas are more likely to smoke. Geographic differences in social norms may therefore provide the key to understanding geographic differences in adolescent smoking.

### 4.5 Pathways linking place to smoking: adolescent-specific factors

Pearce et al. (2012) discuss the pathways linking place-based influences on adult smoking behaviour, in particular in identifying the causes of area-level socioeconomic inequalities in smoking. These include social networks, social capital, social practices, tobacco retailing and advertising. Similar pathways can be theorised to explain geographic differences in adolescent smoking, considering, in addition, adolescent-specific influential factors. For example, issues related to access to tobacco may differ by rurality in terms of how and from whom cigarettes are acquired. In rural areas, there are more adult figures living within the home (Scottish Executive, 2004) who may be influential in socialization of smoking behaviour. Furthermore, a lack of affordable and single occupancy housing in rural areas (Satsangi and Crawford, 2009) may result in older siblings living at home for longer than in urban areas. In sparse rural communities, peer groups are also be more likely to include adolescents of differing ages (Howe, 2010), which might aid supply of cigarettes. Furthermore, peer differences exist between urban and rural areas, concerning issues of identity and belonging (Hendry et al., 2002); smaller peer groups and less choice of friendship groups which may result in greater peer pressure in rural areas to engage in risk behaviours such as smoking. This is in line with Stead et al.'s (2001) urban study of adult smoking, which concluded that prevalence was particularly high in tight-knit isolated communities.

### 4.6 Limitations and recommendations

A minimum of 300 persons per unit area was optimal for geographic comparisons. Even after imputation, the sample sizes achieved were generally lower than 300 for accessible rural areas and remote towns when stratified by sex. However a sample size of 300 was required for a proportion of approximately 65/35%. For smaller proportions, e.g. 17% (current smoking), 12% (weekly smoking) and 10% (daily smoking), smaller sample sizes could be used with the same level of precision. For instance, to measure differences in “current smoking”, a sample of 181 would be sufficient in each sex-geography grouping. For weekly and daily smoking, even smaller samples were sufficient and were for the most part achieved. Sample size requirements were therefore largely met in the current study for all but “ever tried smoking”, although this varied by imputation cycle. The impact of achieving a small sample size, and therefore underpowering, would have been minimal and primarily a problem for the ever tried smoking outcome only.

Qualitative research is recommended to understand reasons for geographic differences in smoking reported in the current study. The findings highlight the important role school initiatives could play in tackling smoking, particularly among girls, and the need to consider gender differences within school initiatives.

The study data were collected three years prior to ban on point of sale tobacco displays in large outlets (areas of 280 m^2^ or more), implemented in April 2013, and extending to small outlets in April 2015 (Primary Medical Services (Scotland) Act (2010)). The aim of the tobacco display ban is to add to the existing cultural shifts in the perceived acceptability of smoking and to reduce uptake of smoking. As size of retail outlet differs by geography (Dawson et al., 2007), this staggered approach is likely to have a more immediate impact on smoking in some geographies than others. The current study therefore gives a measure of adolescent smoking inequalities four years following the smoking ban in public places, and a baseline indication of inequalities prior to the tobacco display ban. Monitoring the impact of this initiative on geographic and socioeconomic inequalities is recommended.

## Figures and Tables

**Table 1:** Definition of the urban-rural classification used.

Rural classification	Description^a^	% of study sample	% of Scottish population^a^
4 cities	Settlements with population over 125,000 (i.e. Aberdeen, Dundee, Glasgow, and Edinburgh)	24.1	38.9
Other urban	Other settlements with population over 10,000	23.7	30.3
Accessible towns	Settlements with population between 3 and 10,000 and within a 30 min drivetime of a settlement of 10,000 or more	10.5	8.6
Remote towns	Settlements with population between 3 and 10,000 and more than 30 min drivetime of a settlement of 10,000 or more	9.4	4.1
Accessible rural	Settlements with population less than 3000 and within a 30 min drivetime of a settlement of 10,000 or more	14.6	11.2
Remote rural	Settlements with population less than 3000 and more than 30 min drivetime from a settlement of 10,000 or more	17.7	7.0

a Source: Scottish Government, 2008.

**Table 2:** Prevalence of smoking by gender, family affluence, school type and rurality, [*n*] % (s.e.); complete-case dataset, *N* = 2692.

Variable	Tried smoking	Current smoking	Weekly smoking	Daily smoking
Gender				
Male	[435] 34.3 (1.5)	[172] 13.6 (1.1)	[126] 9.9 (0.9)	[100] 7.9 (0.8)
Female	[606] 42.8 (1.6)	[267] 18.9 (1.2)	[200] 14.1 (1.0)	[151] 10.7 (0.9)

Family Affluence Scale				
Low FAS	[373] 40.3 (1.8)	[169] 18.3 (1.2)	[130] 14.1 (1.2)	[104] 11.2 (1.1)
Medium FAS	[346] 38.5 (1.6)	[153] 7.0 (1.4)	[116] 12.9 (1.2)	[86] 9.6 (1.1)
High FAS	[322] 37.4 (1.7)	[117] 13.6 (1.3)	[80] 9.3 (1.0)	[61] 7.1 (0.9)

School type				
State school	[1009] 39.1 (1.2)	[421] 16.3 (0.9)	[317] 12.3 (0.8)	[248] 9.6 (0.7)
Independent school	[32] 31.4 (4.9)	[18] 17.6 (3.8)	[9] 8.8 (3.2)	[3] 2.9 (1.6)

Rurality				
4 Cities	[212] 32.8 (2.6)	[88] 13.6 (1.5)	[69] 10.6 (1.4)	[48] 7.4 (1.3)
Other urban	[243] 38.1 (2.3)	[117] 18.4 (2.0)	[90] 14.2 (1.5)	[67] 10.5 (1.3)
Accessible towns	[126] 44.7 (3.4)	[45] 16.0 (2.5)	[33] 11.7 (2.4)	[29] 10.3 (2.3)
Remote towns	[107] 42.6 (4.1)	[38] 15.1 (2.3)	[26] 10.4 (1.9)	[22] 8.8 (2.0)
Accessible rural	[144] 36.6 (2.2)	[62] 15.8 (1.8)	[41] 10.4 (1.7)	[35] 8.9 (1.6)
Remote rural	[209] 44.1 (2.5)	[89] 18.8 (2.1)	[67] 14.1 (1.7)	[50] 10.5 (1.6)

Deprivation (SIMD^a^ quintiles)				
SIMD 1 (most deprived)	[117] 36.9 (3.2)	[53] 16.7 (2.4)	[48] 15.1 (2.2)	[39] 12.3 (2.2)
SIMD 2	[201] 48.6 (2.3)	[86] 20.7 (2.2)	[62] 14.9 (1.8)	[52] 12.5 (1.8)
SIMD 3	[235] 38.3 (1.8)	[116] 18.9 (1.6)	[95] 15.5 (1.6)	[71] 11.6 (1.4)
SIMD 4	[279] 39.5 (2.1)	[106] 15.1 (1.6)	[69] 9.8 (1.1)	[51] 7.2 (1.0)
SIMD 5 (least deprived)	[209] 33.0 (2.0)	[78] 12.3 (1.2)	[52] 8.2 (1.2)	[38] 6.0 (0.9)

Clustering by school is accounted for in the calculation of SEs.

a SIMD income domain.

**Table 3:** Multilevel logistic models for categorical boys' smoking outcomes, MCMC^a^ estimation, odds ratios and credible intervals.

Fixed effects	Tried smoking	Current smoking	Weekly smoking	Daily smoking
Age	1.21 (0.99, 1.47)	1.20 (0.73, 1.97)	1.48 (0.97, 2.24)	1.01 (0.65, 1.55)

Family Affluence Scale (Ref: High FAS)				
Medium FAS	0.84 (0.62, 1.13)	1.10 (0.72, 1.67)	1.28 (0.79, 2.06)	1.09 (0.63, 1.88)
Low FAS	0.79 (0.58, 1.07)	0.87 (0.56, 1.36)	1.08 (0.64, 1.80)	1.13 (0.65, 1.97)

Deprivation (Ref: SIMD^b^ 5 (least deprived))				
SIMD 4	1.25 (0.87, 1.81)	1.41 (0.83, 2.41)	1.06 (0.56, 2.00)	0.81 (0.39, 1.65)
SIMD 3	1.07 (0.72, 1.58)	2.00 (1.16, 3.44)^d^	2.33 (1.29, 4.21)^d^	1.88 (0.98, 3.62)
SIMD 2	2.51 (1.63, 3.87)^d^	2.45 (1.36, 4.43)^d^	2.02 (1.04, 3.94)^d^	1.79 (0.86, 3.70)
SIMD 1 (most deprived)	1.43 (0.88, 2.35)	1.39 (0.68, 2.81)	1.44 (0.66, 3.11)	1.25 (0.53, 2.93)

School type (ref: State school)				
Independent school	0.65 (0.24, 1.73)	1.25 (0.39, 4.02)	0.69 (0.13, 3.81)	0.95 (0.16, 5.49)

Rurality (Ref: 4 cities)				
Other urban	0.88 (0.57, 1.35)	1.08 (0.60, 1.95)	1.03 (0.53, 1.99)	1.01 (0.46, 2.22)
Accessible towns	1.25 (0.76, 2.07)	0.95 (0.46, 1.94)	0.77 (0.35, 1.71)	1.14 (0.47, 2.79)
Remote towns	0.72 (0.41, 1.27)	0.67 (0.30, 1.50)	0.64 (0.26, 1.56)	0.76 (0.28, 2.08)
Accessible rural	1.04 (0.65, 1.66)	0.93 (0.48, 1.78)	0.64 (0.29, 1.41)	0.70 (0.28, 1.70)
Remote rural	1.21 (0.74, 1.97)	0.74 (0.37, 1.49)	0.74 (0.34, 1.61)	0.84 (0.34, 2.04)

Random effects				
Level 1 (child) variance^c^	1	1	1	1
Level 2 (school) variance	0.085 (0.101)	0.433 (0.259)	0.122 (0.198)	0.332 (0.299)
Level 3 (Education authority) variance	0.103 (0.084)	0.055 (0.079)	0.278 (0.230)	0.267 (0.277)

a Via Markov chain Monte Carlo (MCMC); estimates are based on a chain of length of 50,000 following a burn-in of 15,000.

b SIMD income domain.

c Variance at the child level is constrained to 1.

d 95% Confidence Intervals are above or below 1.

**Table 4:** Multilevel logistic models for categorical girls' smoking outcomes, MCMC^a^ estimation, odds ratios and credible intervals.

Fixed effects	Tried smoking	Current smoking	Weekly smoking	Daily smoking
Age	1.63 (1.06, 2.53)^d^	1.14 (0.83, 1.56)	1.50 (1.14, 1.98)^d^	1.14 (0.72, 1.82)

Family Affluence Scale (Ref: High FAS)				
Medium FAS	1.20 (0.90, 1.60)	1.43 (0.96, 2.12)	1.44 (0.94, 2.19)	1.42 (0.88, 2.29)
Low FAS	1.29 (0.96, 1.73)	1.70 (1.15, 2.52)^d^	1.59 (1.04, 2.43)^d^	1.52 (0.94, 2.44)

Deprivation (Ref: SIMD^b^ 5 (least deprived))				
SIMD 4	1.35 (0.95, 1.93)	1.21 (0.77, 1.92)	1.30 (0.76, 2.22)	1.56 (0.82, 2.96)
SIMD 3	1.55 (1.06, 2.26)^d^	1.72 (1.08, 2.76)^d^	2.05 (1.20, 3.49)^d^	2.33 (1.24, 4.38)^d^
SIMD 2	1.88 (1.23, 2.88)^d^	1.93 (1.14, 3.24)^d^	2.10 (1.16, 3.79)^d^	3.00 (1.51, 5.93)^d^
SIMD 1 (most deprived)	1.72 (1.05, 2.81)^d^	2.30 (1.25, 4.22)^d^	3.14 (1.63, 6.05)^d^	4.61 (2.18, 9.75)^d^

School type (ref: State school)				
Independent school	1.36 (0.55, 3.38)	2.07 (0.75, 5.73)	1.31 (0.42, 4.07)	–

Rurality (Ref: 4 Cities)				
Other urban	1.45 (0.92, 2.27)	1.61 (0.94, 2.77)	1.33 (0.74, 2.37)	1.62 (0.82, 3.19)
Accessible towns	1.93 (1.15, 3.24)^d^	1.36 (0.71, 2.61)	1.28 (0.65, 2.54)	1.83 (0.85, 3.94)
Remote towns	2.48 (1.40, 4.39)^d^	1.52 (0.77, 2.99)	1.02 (0.48, 2.17)	1.64 (0.72, 3.73)
Accessible rural	1.44 (0.90, 2.30)	1.64 (0.91, 2.94)	1.53 (0.80, 2.92)	2.58 (1.24, 5.36)^d^
Remote rural	2.10 (1.29, 3.43)^d^	2.66 (1.50, 4.73)^d^	2.29 (1.25, 4.20)^d^	3.00 (1.48, 6.08)^d^

Random effects				
Level 1 (child) variance^c^	1	1	1	1
Level 2 (school) variance	0.330 (0.126)	0.299 (0.162)	0.184 (0.158)	0.055 (0.095)
Level 3 (Education authority) variance	0.062 (0.075)	0.055 (0.077)	0.059 (0.079)	0.131 (0.143)

a Via Markov chain Monte Carlo (MCMC); estimates are based on a chain of length of 50,000 following a burn-in of 15,000.

b SIMD income domain.

c Variance at the child level is constrained to 1.

d 95% Confidence Intervals are above or below 1.

**Table 5:** Multilevel logistic models for categorical boys' and girls' smoking outcomes after imputation, adjusting for age, FAS, SIMD, School type and rurality, MCMC^a^ estimation, odds ratios and credible intervals.

Fixed effects	Tried smoking	Current smoking	Weekly smoking	Daily smoking
**Boys**				
Deprivation (Ref: SIMD^b^ 5 (least deprived))				
SIMD 4	1.22 (0.85, 1.77)	1.23 (0.76, 1.99)	1.07 (0.62, 1.84)	0.92 (0.47, 1.78)
SIMD 3	1.13 (0.77, 1.67)	1.64 (0.99, 2.74)	1.89 (1.07, 3.32)*	1.72 (0.92, 3.21)
SIMD 2	2.25 (1.50, 3.38)*	2.06 (1.19, 3.56)*	1.94 (1.06, 3.53)*	1.86 (0.97, 3.59)
SIMD 1 (most deprived)	1.58 (0.97, 2.56)	1.69 (0.87, 3.29)	1.87 (0.90, 3.86)	1.82 (0.82, 4.03)

Random effects				
Level 1 (child) variance^c^	1	1	1	1
Level 2 (school) variance	0.178 (0.110)	0.430 (0.173)	0.179 (0.161)	0.220 (0.209)
Level 3 (Education authority) variance	0.137 (0.098)	0.079 (0.098)	0.240 (0.177)	0.324 (0.228)
**Girls**				
Family Affluence Scale (Ref: High FAS)				
Medium FAS	1.22 (0.95, 1.57)	1.45 (1.05, 1.99)*	1.47 (1.02, 2.10)*	1.53 (1.01, 2.31)*
Low FAS	1.26 (0.97, 1.64)	1.63 (1.18, 2.25)*	1.66 (1.15, 2.39)*	1.56 (1.03, 2.35)*

Deprivation (Ref: SIMD^b^ 5)				
SIMD 4	1.26 (0.90, 1.78)	1.18 (0.75, 1.83)	1.27 (0.77, 2.10)	1.43 (0.79, 2.58)
SIMD 3	1.49 (1.05, 2.12)*	1.60 (1.03, 2.49)*	1.85 (1.10, 3.10)*	2.02 (1.12, 3.65)*
SIMD 2	1.68 (1.12, 2.54)*	1.73 (1.06, 2.82)*	1.91 (1.07, 3.42)*	2.51 (1.32, 4.80)*
SIMD 1 (most deprived)	1.77 (1.15, 2.73)*	2.33 (1.34, 4.04)*	3.08 (1.69, 5.62)*	3.98 (2.06, 7.69)*

School type (ref: State school)				
Independent school	1.46 (0.72, 2.96)	2.42 (1.17, 5.02)*	1.45 (0.62, 3.39)	0.08 (0.01, 1.11)

Rurality (Ref: 4 Cities)				
Other urban	1.30 (0.84, 2.01)	1.33 (0.80, 2.24)	1.02 (0.58, 1.78)	1.25 (0.68, 2.28)
Accessible towns	1.64 (1.01, 2.66)*	1.21 (0.64, 2.26)	1.03 (0.51, 2.05)	1.49 (0.69, 3.19)
Remote towns	2.07 (1.19, 3.59)*	1.34 (0.69, 2.58)	0.91 (0.43, 1.91)	1.50 (0.68, 3.30)
Accessible rural	1.28 (0.82, 1.99)	1.45 (0.82, 2.54)	1.21 (0.65, 2.26)	1.97 (1.02, 3.80)*
Remote rural	1.73 (1.04, 2.87)*	1.99 (1.08, 2.87)*	1.67 (0.90, 3.11)	2.16 (1.13, 4.11)*

Random effects				
Level 1 (child) variance^c^	1	1	1	1
Level 2 (school) variance	0.347 (0.108)	0.328 (0.136)	0.306 (0.166)	0.128 (0.135)
Level 3 (Education authority) variance	0.066 (0.070)	0.034 (0.046)	0.056 (0.071)	0.081 (0.087)

^∗^Significant at 95% level.

a Via Markov chain Monte Carlo (MCMC); estimates are based on a chain of length of 50,000 following a burn-in of 15,000.

b SIMD income domain.

c Variance at the child level is constrained to 1.
